# Antioxidant–Anti-Inflammatory Evaluation of a Polyherbal Formula

**DOI:** 10.3390/ph15020114

**Published:** 2022-01-18

**Authors:** Alice Grigore, Virginia Vulturescu, Georgeta Neagu, Paul Ungureanu, Minerva Panteli, Iuksel Rasit

**Affiliations:** National Institute for Chemical-Pharmaceutical Research and Development-ICCF Bucharest, Calea Vitan, No. 112, 3rd District, 031299 Bucharest, Romania; georgetaneagu2008@gmail.com (G.N.); paulungureanu@yahoo.com (P.U.); minerva.panteli@gmail.com (M.P.); iuksel_rasit@yahoo.com (I.R.)

**Keywords:** *Rosmarinus officinalis*, *Populus nigra*, ICAM-1, DPPH

## Abstract

Most disease—both acute and chronic—results from inflammation, and reactive oxygen species (ROS) are considered some of the strongest stimuli of inflammation. Many studies reported the traditional use of herbal species for treating inflammation, especially when ROS are involved. The present study aims to demonstrate the antioxidant–anti-inflammatory effects of a patented preparation based on *Populus nigra* and *Rosmarinus officinalis* extracts and to highlight its applicative potential; the formula was characterized by HPTLC and HPLC and in-vitro studies were conducted on TNF-α-stimulated HUVECs. The antioxidant activity of the formula was determined by DPPH assay and the phosphomolybdenum method; to assess in-vivo anti-inflammatory activity, a rat paw edema model was used; the formula contains high amounts of polyphenols. It exhibited scavenging activity of 50–85% at 1–10 mg/mL, it inhibited nitrite production and ICAM-1 expression in TNF-α-stimulated endothelial cell cultures dose-dependently, at a maximum of 58.7% at the maximum dose administered and exerted an obvious anti-inflammatory effect in vivo, settling early and decreasing at 180 min; a new herbal bioactive product was presented with promising therapeutic potential that can be an adjunct to conventional therapies for diseases based on oxidative stress and inflammation.

## 1. Introduction

Most disease—both acute and chronic—results from inflammation. The cascade of inflammation is the body‘s unique mechanism of maintaining its integrity in response to various injuries [1]. Phagocytes, including polymorphonuclear leukocytes (neutrophils, eosinophils) and mononuclear cells (macrophages, lymphocytes), excessively produce reactive oxygen species (ROS), which play an important role in the hosts’ defense mechanisms against microorganisms. In addition to this defensive effect, excessive ROS production disrupts cellular functions by altering tissues and increasing inflammatory status. ROS are considered some of the strongest stimuli of inflammation because they stimulate the monocyte/macrophage line, increasing the production of proinflammatory cytokines, such as tumor necrosis factor α (TNF-α), interleukin (IL)-8 and IL-1β, which are some of the most important factors of the inflammatory response. Thus, the inhibition of ROS production represents one means of evaluating the anti-inflammatory potentials of some compounds.

Many studies reported the traditional use of herbal species for treating inflammation, especially when ROS are involved. Poplar species belonging to the Salicaceae family are widely used for this purpose: male inflorescence of *Populus tomentosa* Carr. or *Populus canadensis* Moench [2], *Populus deltoides* W. Bartram ex Marshall leaf extract [3], *Populus nigra* L. and *Populus × berolinensis* Dipp. leaf-buds (Populi gemmae) [4]. The extracts of *Populus alba* L. leaf and shoots demonstrated substantial antioxidant activities in vivo in a multicellular model organism, *Caenorhabditis elegans,* mainly due to components such as salicin, isorhamnetin-3-*O*-β-D-rutinoside and gallocatechin [5].

The traditional use of *Rosmarinus officinalis* L. (Lamiaceae family) as an effective remedy for pain relief and inflammatory disorders is also well documented. Rosemary extracts proved to alleviate rheumatism and asthma [6], acnes [7], chronic liver disease [8], inflammatory bowel disease [9], neuro-inflammation [10] and arthritis [11,12].

The present study aims to demonstrate the antioxidant–anti-inflammatory effects of a patented preparation based on *Populus nigra* and *Rosmarinus officinalis* extracts and to highlight its applicative potential as a dietary supplement, complementary medicine or cosmetic ingredient.

## 2. Results

### 2.1. Analytical Studies

#### 2.1.1. Qualitative Analysis by High-Performance Thin-Layer Chromatography (HPTLC)

**Figure 1:**
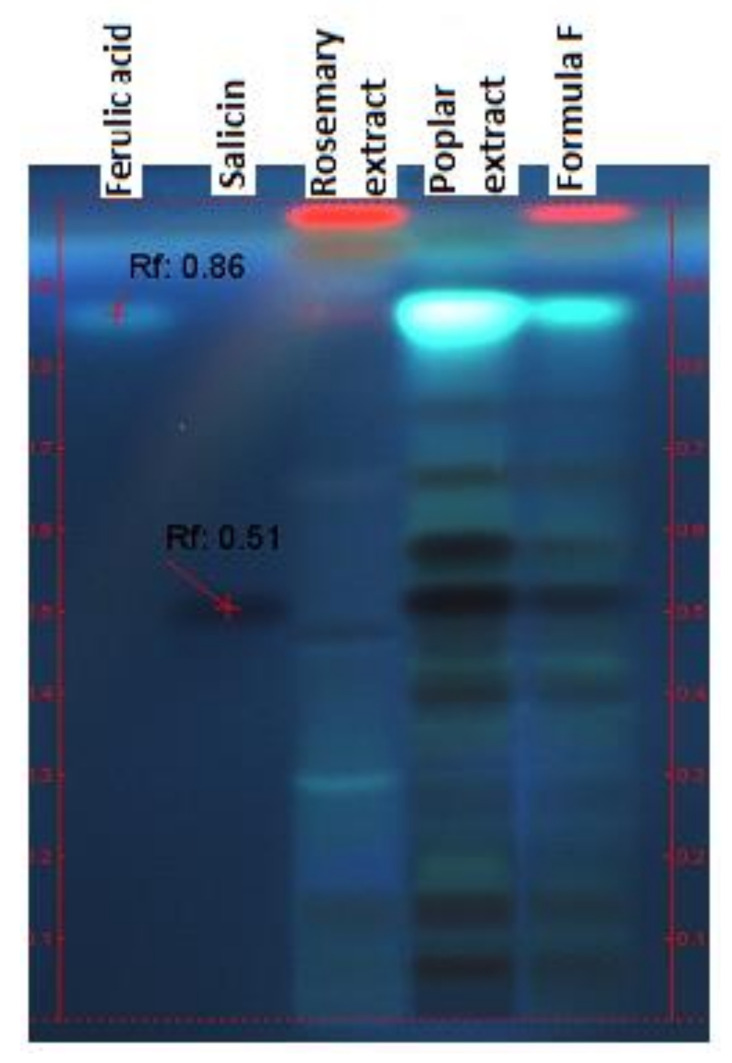
Identification of salicin by HPTLC, derivatization with 20% H_2_SO_4_ (UV 366 nm).

**Figure 2:**
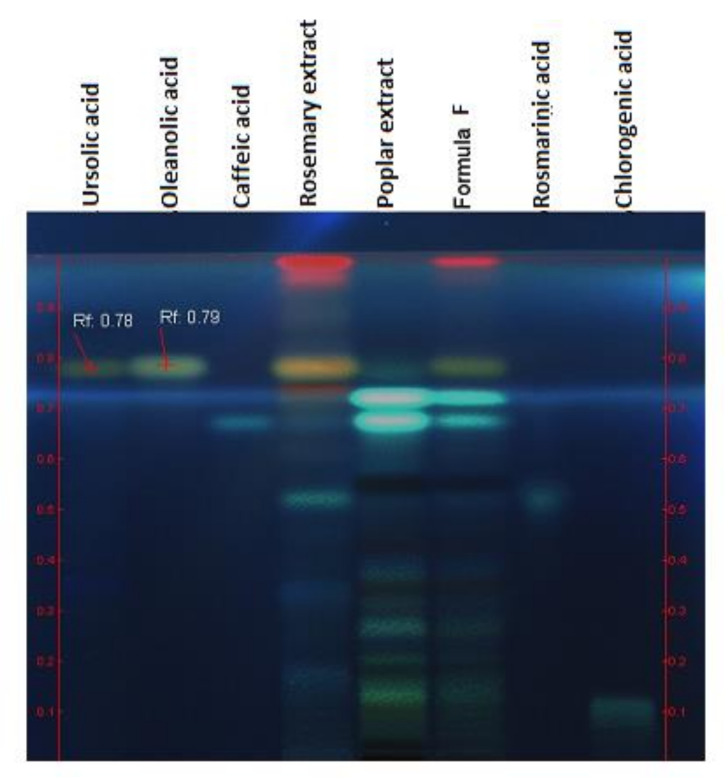
Identification of triterpenic and polyphenolcarboxylic acids; derivatization with *p*-anisaldehyde, UV 366 nm.

HPTLC analysis aimed to highlight the marker compounds in the final formula in comparison with its component extracts. Figure 1 reveals the presence of salicin in the formula and poplar extract. Shown in the second figure (Figure 2), triterpenic (ursolic and oleanolic) and polyphenolcarboxylic (chlorogenic and caffeic) acids were identified in the formula.

#### 2.1.2. Spectrophotometric Analysis of Phenolic Compounds

The content of flavones expressed in rutin and polyphenolcarboxylic acids and expressed in gallic acid was quantified spectrophotometrically, the results being expressed as a percentage (Table 1)

#### 2.1.3. Quantitative Analysis by High-Performance Liquid Chromatography (HPLC)

**Figure 3:**
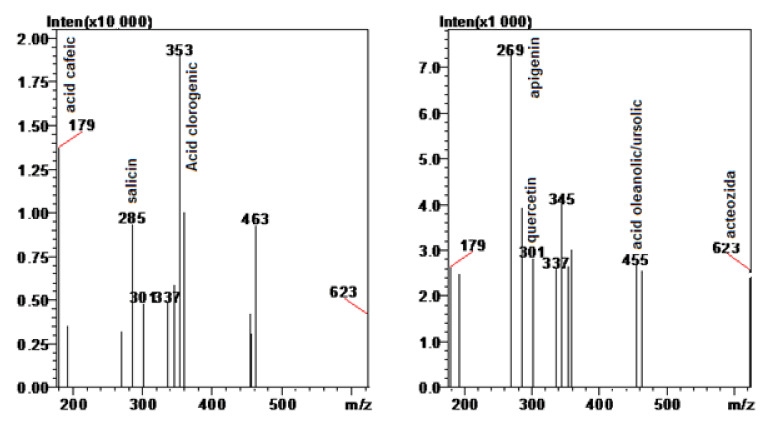
Mass spectra of the formula, normalized for apigenin and chlorogenic acid.

The content of the most representative compounds of the formula are shown in Table 2 and Figure 3. Chromatograms are available as Supplementary Materials (Figures S1–S3).

Rosemary and poplar buds are well known sources of phenolic compounds but the amount and relative contribution of each class of (poly)phenolic compounds from rosemary extracts were reported to be dependent on the extraction procedure and solvent used. Dry leaf decoction was found to be the next best extraction method for rosemary after Soxhlet, yielding significantly higher caffeic acid, rosmarinic acid, carnosol, carnosic acid and flavonoids. Therefore, it was suggested that this might be the best method for large-scale commercial extraction [13]. Our formula was obtained by several phases of extraction, using both maceration and reflux techniques such that the end products have a balanced content of active compounds. Comparing with other studies, the content of rosmarinic acid was lower, but, instead, an important amount of caffeic acid was obtained.

In a previous study, a detailed ultrahigh-performance liquid chromatography–electrospray ionization multiple reaction monitoring tandem mass spectrometry (UHPLC–ESI–MS) analysis of a rosemary fraction was conducted to identify 57 (poly)phenolic compounds belonging to different phenolic groups (24 flavonoids, 5 phenolic acids, 24 diterpenes, 1 triterpenic acid, and 3 lignans) [14].

The amount of phenolic compounds previously reported for other rosemary extracts was quite variable and ranged from ~39.3 mg/g [15] to 523 mg/g [16]. Most of the studies yielded high concentrations of rosmarinic acid, up to 33.49 mg/g, which contributes substantially to explaining the high antioxidant potential of the extracts [13].

Oleanolic acid and ursolic acid, typically present in the triterpenoid fraction of rosemary, were not always detected [14].

The literature data shows that black poplar buds contain flavones (e.g., apigenin and chrysin) and flavanones (e.g., pinocembrin and pinostrobin). Beside this class of phytochemicals, other important representatives are the phenolic compounds, such as caffeic, ferulic acids and their derivatives [17]. Dudonne et al. [18] have shown that aqueous black poplar bud extracts from Bulgaria contain phenolic acids (caffeic, cinnamic, coumaric, ferulic and di-O-methyl caffeic acid), flavonoids (pinocembrin and pinobanksin) and also salicin.

### 2.2. Pharmacological Studies

#### 2.2.1. Total Antioxidant Capacity

Natural products with antioxidant activity have been recognized as valuable tools in the management of oxidative/nitrosative stress-induced pathologies [19]. Plants that contain certain amounts of polyphenols and flavonoids are perfect sources of antioxidants [20,21], but some studies infirmed this claim [22]. In other studies, the total phenolic content and flavonoid content did not correlate well with the results from the 1,1-diphenyl-2-picrylhydrazyl (DPPH) test.

As antioxidants may have different mechanisms of action, two types of assays were chosen for assessing the antioxidant potential of formula F: a reducing antioxidant power assay (phosphomolybdic acid method) and scavenging activity assays (DPPH assay).

**Figure 4:**
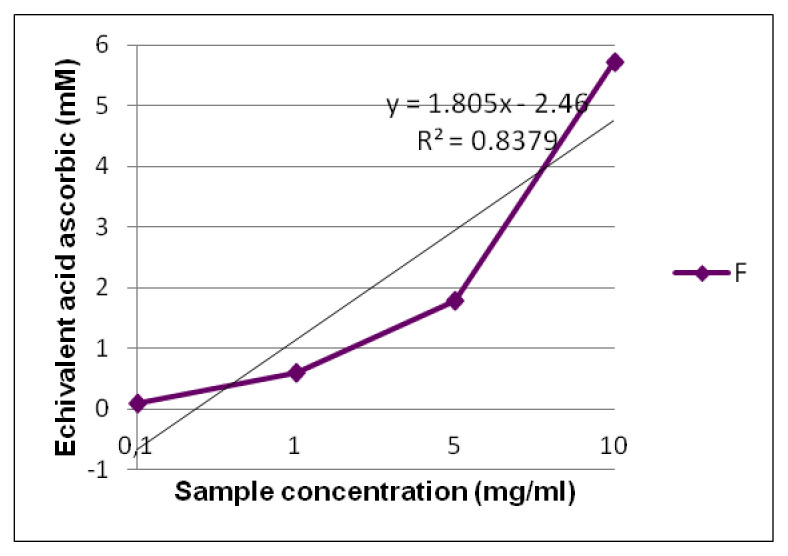
The total antioxidant capacity of formula F evidenced by the phosphomolybdic acid method.

Total antioxidant capacity was performed by the phosphomolybdic acid method based on the reduction of Mo (VI) to Mo (V) by a compound with antioxidant potential with the formation of a green phosphate/Mo (V) complex having maximum absorption at 695 nm. The method is used successfully to quantify vitamin E and it has been extended to determine the antioxidant capacities of plant extracts. It is a quantitative method, since the antioxidant activity is expressed as a number of ascorbic acid equivalents. Formula F exerts an antioxidant effect of, at maximum, 5.72 mM equivalent ascorbic acid at a concentration of 10 mg/mL, which decreases to 0.6 mM equivalent ascorbic acid at 1 mg/mL (Figure 4).

#### 2.2.2. DPPH Scavenging Assay

DPPH assay is widely used for determining scavenging potential. The antioxidant molecules present in plant extracts transform DPPH radicals into stable molecules by transferring hydrogen atoms from their molecules and/or electrons from their atoms. This steady conversion results in a decrease in absorbance and indicates the scavenging potential of analytes [23].

**Figure 5:**
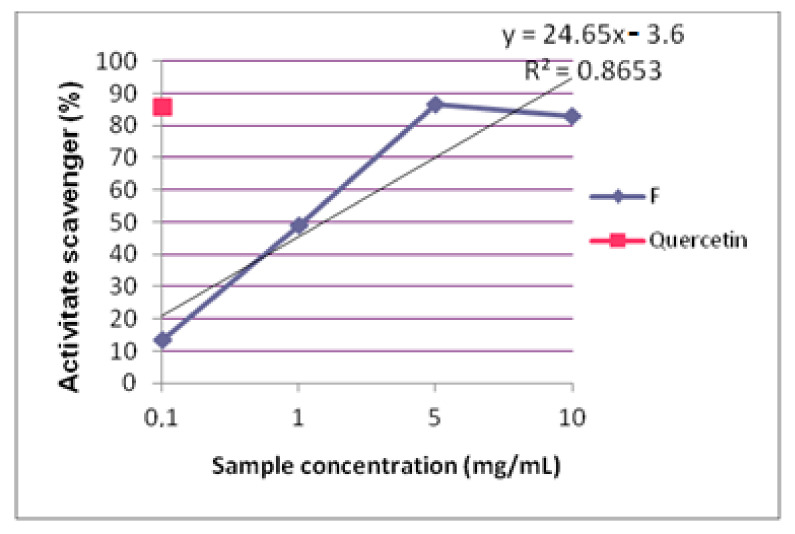
Scavenging activity of formula F.

The evaluation of the scavenging capacity of formula F reveals an antioxidant action of over 50%, even at doses of 1 mg/mL, with an IC50 of 0.97 mg/mL (Figure 5). Studies related to the reducing effect on the radical DPPH of single extracts reported low IC50 values in the case of rosemary hydroalcoholic extract (4.63 µg/mL) [24] and higher values for poplar extract (0.2 mg/mL) [25]. Considering these data, we might expect a synergic action from both extracts in formula F, but our results were closer to those reported in the literature for poplar extract. The yield of phenolic compounds and also their scavenging effects are strongly correlated to extraction method, which could explain the differences between studies.

In one study, compounds derived from rosemary leaves (carnosol, carnosic acid, rosmadial, genkwanin, rosmarinic acid) were analyzed by biophysical techniques on membrane models and it was found that they exert stiffening effects on the plasma membrane that may contribute to antioxidant capacity by preventing the diffusion of free radicals [26].

#### 2.2.3. In-Vitro Assays

##### Evaluation of Nitric Oxide (NO) Production

Multiple experimental results from the last 15 years confirmed that increased NO production by the inducible-nitric oxide synthase (iNOS) pathway induced by proinflammatory cytokines is essential in the pathophysiology of inflammation. The involvement of NO in rat paw edema was established by Ialenti in 1992 [27], who reported a reduction in edema and hyperpermeability at the time of carrageenan coinjection with NOS inhibitors such as N (gamma)-nitro-L-arginine methyl ester (L-NAME) or N (omega)-Monomethyl-L-Arginine Acetate (L-NMMA). Carrageenan injection was later shown to initiate the formation of nitrotyrosine, which was suppressed by iNOS inhibition [28].

A line of human endothelial cells was chosen for the experiment because several phases of the inflammatory process take place in the endothelium and, in addition, nitric oxide has an important role in modulating endothelial tone. Cytotoxicity tests were performed beforehand in order to establish the optimal doses for assays (data not shown).

TNF-α was used as an inflammatory stimulus and two experimental variants were chosen, in which the time of stimulation with TNF-α and the time of administration of the extracts were varied (four concentrations of formula F). In both cases, the samples were administered before their stimulation with TNF-α as a protective agent.

According to the literature data, the protection against NO could be attributed to rosmarinic acid, which has been reported to inhibit the expression of iNOS in Raw 264.7 macrophages [29]. Carnosic acid inhibits NO completely at concentrations of >12.5 µg/mL [30] and carnosol with an IC50 of 9.4 µM [31].

Previous studies showed that, in the case of rosemary extracts, the activity correlates with the content of triterpene compounds, wherein the anti-inflammatory effects were attributed to the inhibition of various stages of the inflammatory reaction–histamine release, cyclooxygenase (COX) and lipoxygenase (LOX) activity, complement and NO production [32].

A comparative study on the antioxidant action of standardized rosemary extracts containing 20% carnosic acid, 40% ursolic acid and 20% rosmarinic acid showed that the extract enriched in ursolic acid demonstrated the weakest antioxidant capacity, the extract enriched in rosmarinic acid was showed more effective oxygen radical absorbance capacity (ORAC) and ferric ion-reducing antioxidant power (FRAP), and the extract enriched in carnosic acid was the most active in an ex-vivo, low-density lipoprotein (LDL) oxidation inhibition model [32].

Our formula is characterized by important amounts of phenolics, namely, caffeic acid, and this class of compounds could be responsible for its antioxidant action.

**Figure 6:**
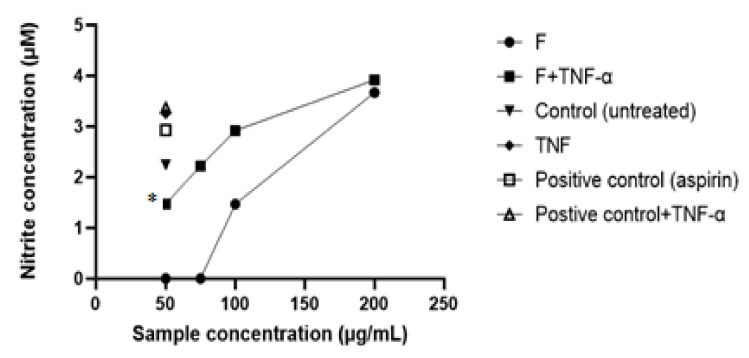
NO inhibitory potential of formula F. Each point indicates the mean ± SD of three measurements. * *p* < 0.05 was considered significantly different compared with the unstimulated group.

The cells were incubated for 2 h with formula and then stimulated with TNF-α for 17 h. In the case of co-stimulation of formula F at a concentration of 50 μg/mL with TNF-α, there is an increase in nitrite production (*p* = 0.031 < 0.05) (Figure 6). The level of nitrites increases with dose of formula F. In general, the trends are similar for cells treated with extract but not stimulated and those treated and stimulated, suggesting that, at high doses, the formula might induce, by itself, NO production in endothelial cells. After a short incubation of the cells with formula and then stimulation with TNF-α, the response of the cells to nitrite production was comparable between stimulated and unstimulated cells. At long-term administration of the extract, the nitrite values obtained were much higher and a clear delimitation could be made between the stimulated cell group and the unstimulated control group. At the highest concentration tested, the formula induced an increased level of nitrite over the TNF-α-treated group, failing to induce a cytoprotective effect against endothelial cell damage.

##### Intercellular Adhesion Molecule (ICAM)-1 Quantification

**Figure 7:**
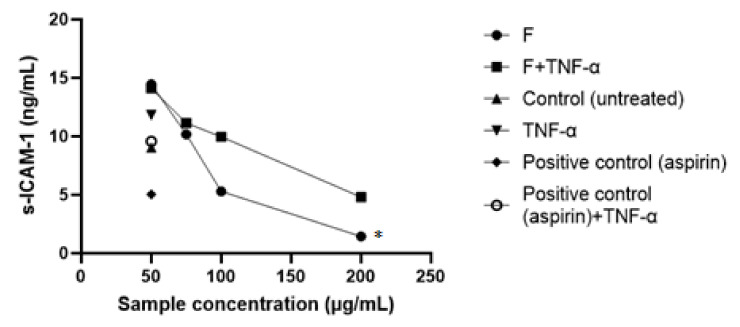
ICAM-1 expression in HUVECs stimulated with TNF-α. Each point indicates the mean ± SD of three measurements. * *p* < 0.05 significantly different compared to unstimulated group.

Evaluation of ICAM-1 expression in the culture of endothelial cells exposed to various concentrations of formula F or controls for 2 h and stimulated further for 17 h with TNF-α was performed by the ELISA method. The results are presented in Figure 7 and represent the average values obtained for each tested lot.

Another experimental model to highlight the anti-inflammatory activity of the extracts was to highlight the production of adhesion molecules in the culture of endothelial cells stimulated with TNF-α. The expression of adhesion molecules on the surface of endothelial cells is one of the initiating events of the inflammatory process. As in the previous experiment, the protective effect of the formula on human umbilical vein endothelial cells (HUVEC) stimulated with an inflammatory stimulus (TNF-α) was investigated, but here the measured parameter was ICAM-1, by an enzyme-linked immunosorbent assay (ELISA). The cells were incubated with four concentrations for 2 h and then stimulated with TNF-α for 17 h; as was shown in the previous experiment, this was predicted to be the combination to which the cells would respond best. In comparison, a blank group (cells incubated with plant extracts but not stimulated with TNF-α) and three control groups (untreated and unstimulated cells, untreated but TNF-α-stimulated cells, as well as TNF-α-stimulated cells treated with acetylsalicylic acid 10 mM), were evaluated.

In the case of unstimulated-but-F-treated cells, ICAM-1 expression increased dose-dependently with extract administration. Statistically significant differences were recorded at the maximum dose administered—200 μg/mL in the case of formula F. Cells stimulated only with TNF-α reached a level of 11.81 ng/mL ICAM-1, comparable with cells protected with plant extract at the minimum concentration of 50 μg/mL. Below the ICAM-1 level produced by the cell control group was formula F at 200 μg/mL. Formula F exerted an inhibitory effect of over 50% only at a dose of 200 μg/mL (*p* = 0.0129 < 0.05), whereas at lower doses the effect was opposite—the stimulation of ICAM-1 secretion. The effect induced by the reference substance, acetylsalicylic acid, was similar to the control groups, both untreated and unstimulated.

Several isolated compounds proved their efficacy in reducing CAMs expression and highlighted the dual effect, both antioxidant and anti-inflammatory; rosmarinic acid reduced the level of reactive oxygen species, H_2_O_2_-dependent VEGF expression and the release of IL-8 by endothelial cells [33], and also the TPA-induced expression of ICAM-1, vascular cell adhesion molecule (VCAM)-1, COX-2 and macrophage inflammatory protein (MIP)-2 [34]. Caffeic acid inhibited TNF-α-induced NF-κB-DNA complex formation and CAMs expression, suggesting its potential role in atherosclerosis diseases [35]. Although the role of salicin and willow bark extract in reducing inflammatory exudates and leukocyte infiltration, as by preventing the increase in cytokines, is well known, it was nonetheless shown that, additionally, other constituents of the extract contributed to its anti-inflammatory activity, e.g., other polyphenolic acids [36].

#### 2.2.4. In-Vivo Assay

Of the many methods used in screening for the anti-inflammatory effects of various drugs, the most commonly used is the method of highlighting the inhibitory effect of edema produced in the hind paw of a rat after the injection of an inflammatory product. We used whole, fresh egg white, which, due to its inflammation being maintained for a relatively short period of time, is optimal for observation [37]. The effect was measured by the computerized plethysmometric method, the method unanimously and currently used in testing systemic anti-inflammatory effects.

The increases in the volume of the inflamed extremity at 30, 60, 120 and 180 min were expressed as a percentages compared with the volumes measured immediately after injection of the irritant (Table 3). The mean values of the measured volumes for each animal and the times of measurement were calculated, as were ± standard deviations, followed by the mean values per batch (Table 4). The differences between the averages of the values obtained in the treated groups and those of the control groups (with or without treatment), were calculated for each measurement moment and the anti-inflammatory indexes were evaluated.

The anti-inflammatory action against the inflammatory agent is manifest if the anti-inflammatory index is higher than 20%. Values lower than this percentage denote the lack of anti-inflammatory action against the inflammatory agent. For the coefficients of variation obtained, the average values of the lots are representative.

(a) 30 min after the edema was caused, a detectable anti-inflammatory effect (>20%) was perceived for formula F and the ASP 100 reference substance.
(b) At 60 min after causing the edema, the samples were above the 20% level.
(c) At 120 min, the samples had an anti-inflammatory effect.
(d) At 180 min there was a marked anti-inflammatory effect of the ASP 100 sample while formula F had no anti-inflammatory effect.

The reference substance, ASP 100, showed an anti-inflammatory effect that was induced early and showed an important effect of preventing inflammation of the irritated rat paw, an effect represented by low values of the volume of the extremity with edema caused throughout the observation period. Formula F showed an obvious anti-inflammatory effect, settling early and diminishing at 180 min.

A classic method, the induction of rat paw edema, was chosen to evaluate the in-vivo anti-inflammatory activity of the extracts. Fresh egg white, which induces the secretion of histamine and 5-hydroxytryptamine, which are involved in both inflammatory and allergic reactions, was used as an inflammatory agent. Histamine exerts proinflammatory effects through vasodilation, effects regulating certain links of the inflammatory reaction (increasing local vascular permeability, stimulating neutrophils and eosinophils through H^2^ receptors, increasing the production of interleukins), but also anti-inflammatory effects (reducing the intensity of inflammatory effects due to the stimulation of H_2_ on the surfaces of some cells, when the amount of histamine released is very high).

The model was also used in other studies, proving the anti-inflammatory effect of various rosemary and poplar extracts. Administration of rosmarinic acid and rosemary extract at the dose of 25 mg/kg reduced paw edema, after 6 hrs, by over 60%, exhibiting a dose–response effect, suggesting that rosmarinic was the main contributor to the anti-inflammatory effect [38]. The anti-inflammatory and antioxidant activities of the rosemary can be attributed—partly, at least—to its content of polyphenolics with the strong possibility of synergistic interactions, as well as metabolic activations [39]. A rosemary aqueous extract rich in phenolic content (196.63 ± 3.09 mg gallic acid equivalents GAE/g E) and flavonoids (2.22 ± 0.09 mg rosemary extract/g E), showed good inhibitory action (44.62% in the third hour for the higher dose. *: *p* < 0.1), close to that of aspirin (ASP) which was used as a standard (31.87% in the third hour for 200 mg/kg aspirin. **: *p* < 0.05) [40].

Additionally, it was shown that an ethanolic extract of *P. nigra* showed a moderate antioxidant activity (40%), but potent anti-inflammatory activity (49.9%) on carrageenan-induced mice paw edema [41].

Statistically significant values (*p* < 0.05) were recorded for formula F and the reference substance at 30 min, 60 min and 120 min. Formula F showed an obvious anti-inflammatory effect, settling early and diminishing at 180 min. The product administered orally at a dose of 250 mg/mL/100 g of body weight (bw) exerted an influence on the early experimentally induced edema initially comparable to that of the reference product, ASP 100, kept constant throughout the experiment in intensity and duration.

## 3. Materials and Methods

### 3.1. Method of Preparation (According to Patent No.RO126280/29.06.2012)

Raw material was purchased from a local store and voucher specimens were deposited at the institute (No. PN3/2020 for *Populus nigra* and RO3/2020 for *Rosmarinus officinalis*).

The process of obtaining the phytotherapeutic product (final formula F) consisted of homogenizing for 30 min and the continuous stirring of five-parts-by-weight Rosmarini herb selective extract, with two-parts-by-weight Populi gemmae selective extract (previously dissolved in 50% ethanol). After drying and grinding, 3.2 g of non-hygroscopic brown-green powder with an aromatic odor and bitter taste were obtained. The detailed technological process has been described in the patent.

Briefly, the *Populus nigra* extract was prepared by extraction of the plant material (dried and ground poplar buds) with 50% ethyl alcohol (plant/solvent ratio = 1/10, *m*/*v*), at the reflux temperature of the solvent, under continuous stirring, for 2 h. The plant material was then subjected to a second extraction with 20% ethyl alcohol (plant material/solvent ratio = 1/8, *m*/*v*) at the reflux temperature of the solvent, under continuous stirring, for 1 h. The two extracts were combined and concentrated at 40 °C until the alcohol was removed, left to settle, then centrifuged to obtain an opalescent extract which was concentrated at 40 °C under reduced pressure and precipitated with methyl alcohol (aqueous solution/solvent ratio = 1/4, *v/v*). The filtrate was concentrated at 40 °C under reduced pressure (72–74 mmHg) to a volume of 100 mL after which it was subjected to liquid–liquid extraction with n-butyl alcohol in a graduated separator funnel, six times in succession with 300 mL of solvent (aqueous solution/solvent ratio = 1/3, *v/v*). The n-butyl alcohol extracts were combined and concentrated at 60 °C under low pressure to obtain the P extract as a thick, strongly aromatic brown residue.

The *Rosmarinus officinalis* extract was prepared by macerating the dried plant material with acetone (plant/solvent ratio = 1/7, *m*/*v*), at room temperature, stirring occasionally, for 3 h. The obtained solution was concentrated at 40 °C under low pressure (72–74 mmHg) up to a smaller volume and filtered. The filtrate was concentrated at 40 °C under low pressure (72–74 mmHg) until only residue remained, after which it was redissolved in 150 mL of methyl alcohol. The plant material was subjected to a new extraction with 20% ethyl alcohol (plant material/solvent ratio = 1/10, *m*/*v*), at the reflux temperature of the solvent, with continuous stirring, for 2 h. After cooling and filtration, a brown, opalescent hydroalcoholic extract was obtained, concentrated at 40 °C under low pressure (72–74 mmHg) up to a volume of 150 mL and reunited with the fraction previously dissolved in methyl alcohol. The precipitate obtained was filtered, dried in an oven at 40 °C and represented the R extract in the form of a brown-greenish, non-hygroscopic powder.

For analytical and in-vitro assays, formula F was used as a stock solution of 500 mg/mL in 50% ethanol.

### 3.2. Analytical Studies

#### 3.2.1. HPTLC

The identification of the main classes of compounds in the final formula was performed by HPTLC, using a Camag HPTLC system (Camag, Muttenz, Switzerland) equipped with Linomat V automatic applicator and WinCats data processing software. The identification of salicylates and polyphenolcarboxylic acids was performed by a thin layer chromatographic method, according to Camag indications [42], and for triterpene acids identification, the method from [43] was used. The marker compounds were analyzed comparatively in the formula and also in the component extracts. Reference compounds were purchased from Sigma-Aldrich (Darmstadt, Germany): salicin, chlorogenic acid, oleanolic acid, caffeic acid, ferulic acid, rosmarinic acid.

#### 3.2.2. Spectrophotometric Analysis of Phenolic Compounds

Flavonoids were dosed by a colorimetric method based on their properties to form chelated complexes colored with trivalent metal ions Al^3+^ + which have a maximum absorption in the VIS at λ = 430 nm [44]. Quantification of the total phenolic compounds expressed in gallic acid was performed by a Folin–Ciocalteu colorimetric method [45], which is based on the formation of colored complexes with maximum absorption in VIS at λ = 750 nm.

#### 3.2.3. HPLC

HPLC determination of the compounds of interest was performed using two methods, one for polyphenolcarboxylic acids and the other for terpenes. The optimal conditions for analysis were determined using specific standards for compounds of interest. Quantitative HPLC analysis of the main components was performed on a HPLC SHIMADZU system, with LC-20AD sp pumps, a DGU-20As Degasser, a CTO-20AC thermostat column and a DAD–MS detector (SPD-M20A diode array and MS Shimadzu). A Kromasil C18 column (100 mm × 4.6 mm, particle size 5 µm) for polyphenolcarboxylic acids and a C8 column (100 mm × 2.1 mm, particle size 3.5 µm) for triterpenes, at 25 °C were used, using a gradient elution. Separation of polyphenols was performed using a mobile phase consisting of an A solution (water acidified with formic acid 1%) and a B solution (methanol acidified with formic acid 1%) at an initial flow rate of 0.1 mL/min; with an injection of 15 μL. Given that a DAD–MS tandem detection system was used, a flow rate of 0.1 mL/min was initially used, with both a mobile-phase flow gradient and a mobile-phase composition gradient being applied. The C18 column was balanced for 1 h before each set of analyses (of both standards and samples) and the total analysis time was 70 min. For the determination of triterpenes content, the above-mentioned column was used and a mobile phase consisting of ammonium formiate (10 mM) and acetonitrile at an initial flow rate of 0.1 mL/min, with an injection of 15 μL. The total duration of this analysis was also 70 min. For DAD detection, the wavelengths for the identification of the compounds of interest were chosen according to the absorption spectrum recorded for each standard. Both the standards and the samples were analyzed under identical conditions, using a mobile phase in a binary gradient.

After DAD determination, mass-spectrometric analysis was performed, using an ESI interface, the ionic fragments of interest being [M-H^−1^]. MS analysis was extremely important in confirming the identification of compounds of interest in the analyzed samples, which have complex contents.

All reference substances (chlorogenic, rosmarinic, caffeic, ferulic, oleanolic acids, quercetin, apigenin, salicin, acteoside) were from Sigma Aldrich (Darmstadt, Germany).

### 3.3. Pharmacological Studies

#### 3.3.1. Total Antioxidant Capacity

Total antioxidant capacity was evaluated by the phosphomolybdic method. The reaction is based on the reduction of Mo (VI) to Mo (V) in the presence of the extract with the formation of a green phosphate/Mo (V) complex at acidic pH, according to [46].

#### 3.3.2. DPPH Scavenging Assay

DPPH is a stable organic nitrogen radical having a maximum UV–VIS absorption at 515 nm. The DPPH alcoholic solution was contacted with the sample solution; after reduction, the color of the solution faded; the progress of the reaction was monitored by reading the absorbance at 517 nm for 30 min or until the absorbance stabilized. The remaining DPPH percentage was calculated as follows: % inhibition = (absorbing control-absorbing test)/absorbing control × 100%. EC50 is defined as the concentration that decreases the initial DPPH concentration by 50%.

#### 3.3.3. In-Vitro Assays

HUVEC were cultured on 96-well plates 5 × 10^4^ cells/well in RPMI 1640 medium (Sigma-Aldrich, Darmstadt, Germany), supplemented with 10% fetal bovine serum (Sigma-Aldrich, Darmstadt Germany) and 1% antibiotic solution penicillin, streptomycin, neomycin (Sigma-Aldrich, Darmstadt, Germany) and incubated at 37 °C with 5% CO_2_. When reaching confluence, the plant extracts and the inflammatory stimulus were applied as follows:

-  Formula F was solubilized in the culture medium and applied in concentrations of 50, 75, 100 and 200 μg/mL (in duplicate) and maintained at 37 °C with 5% CO_2_ for 2 h
-  After the incubation time elapsed in the presence of the formula, the cells were stimulated with 50 ng/mL TNF-α (human, recombinant TNF-α, Alexis Biochemicals, Lausen, Switzerland) dissolved in serum medium and incubated at 37 °C with 5% CO_2_ for 17 h; in parallel, control groups were settled: formula F alone; untreated control; ASP 1µM; ASP+ TNF-α; TNF-α 50 ng/mL
-  After the incubation time elapsed in the presence of the inflammatory stimulus, the ICAM and NO levels were assessed.

##### Evaluation of NO Production

The evaluation of NOS activity reflected in the degree of NO production in endothelial cell culture was performed using the Griess colorimetric test. NO product was determined indirectly by quantifying the total nitrite levels in the samples according to manufacturer instructions (Alexis Biochemicals, Lausen, Switzerland). Briefly, the determination was performed in a standard 96-well plate. Fifty-microliter samples (formula F in concentrations of 50–200 µg/mL; formula F + 50 ng/mL TNF-α) or standards (untreated control, ASP 1 µM; ASP+ TNF-α; TNF-α 50 ng/mL) were applied in duplicate to each well, 50 μL of Griess reagent was rapidly added (Alexis, Switzerland) and then the plate was shaken for 5 min with a shaker and further incubated for another 5 min at room temperature, protected from light and without agitation. The absorbance was read at 595 nm using a Chameleon LKB 5100 plate reader. The results were reported on a standard sodium-nitrite curve.

##### ICAM-1 Quantification

The in-vitro model was described above. Determination of ICAM-1 production was performed by a colorimetric method using the ICAM-1 (soluble) Human ELISA kit (Invitrogen, Waltham, MA, USA).

#### 3.3.4. In-Vivo Assay

The anti-inflammatory effect of plant extracts was evaluated by rat paw edema assay [47]. Wistar rats, of both sexes, weighing 150–200 g provided by the Cantacuzino Institute Biobase were used for the study. Lots of equal numbers of animals (*n* = 3) were established for each test sample or reference product. The animals were checked at the reception and kept for a period of 7 days for acclimatization, with observation of the clinical condition, behavior and food consumption. They were weighed at the reception and after the acclimatization period, with the selection for testing of animals with weight variations less than 10% of the initial weight. The animals were housed in appropriate cages, in small groups of the same sex. The temperature of the experimental chamber was 22 ± 2 °C and the relative humidity was 50–60%. The lighting was artificial, alternately 12-h light and 12-h dark. The animals received standardized food and water ad libitum. Twelve hours before the test, they were kept at the post and allowed access to water. The measurement of the inflamed segments volumes and their subsequent evolution was performed with the help of a U40 Basile-Italy computerized plethysmometer 7140 and the CUB dedicated software program for data acquisition and storage. The test samples and the reference substance were administered orally by intragastric gavage 1 h before the injection of the inflammatory substance (whole, fresh egg white).

The animals were injected subcutaneously with a volume of 0.1 mL of the inflammatory agent on the plantar surface of the right hind limb.

The volume of the right hind limb was measured plethysometrically prior to injection with the inflammatory agent, which represented the “0” moment of the measurements. The volume of the extremity was measured repeatedly, at 30, 60, 120 and 180 min.

In order to perform the readings, the inflamed extremity was visually marked at the level of the lateral ankle, then it was introduced into the immersion liquid of the measuring cell of the device to this level, with the performance of three measurements for each animal and for each moment, established according to the working protocol, with data storage facilitated by the plethysmometer software. The samples were administered orally by intragastric gavage, as such, at a volume of 1 mL/100 g of bw. Powder samples, after suspension with Tween 80, were administered at a concentration of 250 mg/mL, at 1 mL/100 g of bw.

The reference product (acetylsalicylic acid/aspirin) (Zentiva, Bucharest, Romania) was administered in the same way, in the form of a suspension in two doses of 100 mg/kg of bw at a volume of 1 mL/100 g of bw. The increase in the volume of the inflamed extremity at 30, 60, 120 and 180 min was expressed as a percentage compared to the volume measured immediately after injection of the irritant.

The mean values of the measured volumes for each animal and moment of measurement were calculated, as were ± standard deviations and the mean values per batch.

The differences between the averages of the values obtained in the treated groups and those of the control groups (with or without treatment) were calculated for each measurement moment and their anti-inflammatory indexes were evaluated.

### 3.4. Ethical Statement

All animal studies were approved by the Ethical Committee of the National Institute of Chemical–Pharmaceutical Research and Development (no. 52/17.11.2020) and carried out according to the National Medicine Agency regulations, Directive 2010/63/EU revising Directive 86/609/EEC on the protection of animals used for scientific purposes and Law no. 43/2014 on the protection of animals used for scientific purposes issued by the Romanian Parliament.

### 3.5. Statistical Analysis

In our statistical analysis the paired Student’s *t*-test was used. The results were expressed as means ± S.D. of at least three independent experiments. *p* < 0.05 was considered to be statistically significant. All analysis were performed with GraphPad Prism (version 9.12; GraphPad Software Inc.; San Diego, CA, USA).

## 4. Conclusions

Given the role of inflammation in the pathogenesis and/or prognosis of illness, we require a change in our approaches to illness and disease prevention. Chronic inflammatory diseases such as rheumatoid arthritis, psoriatic arthritis, inflammatory bowel disease and obstructive pulmonary disease are debilitating diseases that affect a large segment of the population. It was shown that metabolic diseases, such as type 2 diabetes and some cardiovascular diseases such as atherosclerosis, can be considered inflammatory diseases in their natures and origins. Hence, inflammation seems to be the root of many chronic diseases and the need to develop effective drugs thereagainst has become imperative.

We proposed a natural product based on rosemary and poplar extracts able to exhibit both antioxidant and anti-inflammatory activities that could be used as complementary treatment in various ailments having inflammation as a main symptom.

## Figures and Tables

**Table 1:** Content of total polyphenols and flavones of formula F, determined spectrophotometrically.

Sample	Total Polyphenols Expressed as Gallic Acid (%)	Flavones Expressed as Rutin (%)
Formula F	12.12 ± 0.542	6.14 ± 0.384

**Table 2:** The content of compounds of interest of formula determined by HPLC.

Compound	Formula F
chlorogenic acid	14.85 ± 0.25 mg/g
caffeic acid	62.16 ± 0.12 mg/g
ferulic acid	13.94 ± 0.14 mg/g
rosmarinic acid	8.91 ± 0.27 mg/g
quercetin	15.4 ± 0.24 mg/g
apigenin	0.342 ± 0.18 mg/g
salicin	7.7 ± 0.13 mg/g
oleanolic acid	1.86 ± 0.12 mg/g
acteoside	12.54 ± 0.2 mg/g

**Table 3:** Anti-inflammatory activity of formula F on egg white-induced rat paw edema.

Time (min)	30	60	120	180
% inhibition of paw volume				
formula F	38.65	35.42	36.64	12.76
ASP 100	42.98	58.20	64.25	54.40

**Table 4:** Average paw size (mL) at 0, 30 min, 1 h, 2 h and 3 h after causing edema.

Time (min)	0	30	60	120	180	30	60	120	180
Groups	Average Paw Size (mL) ± SD					Average Rise in Paw Volume (mL) ± SD			
control	1.523 ± 0.137	2.830 ± 0.314	2.895 ± 0.290	2.843 ± 0.298	2.547 ± 0.244	1.307 ± 0.177	1.372 ± 0.153	1.32 ± 0.161	1.024 ± 0.107
formula F	1.148 ± 0.044	* 1.949 ± 0.111	* 2.033 ± 0.025	* 1.983 ± 0.085	2.040 ± 0.026	* 0.801 ± 0.071	* 0.885 ± 0.019	* 0.835 ± 0.041	0.892 ± 0.018
ASP 100	1.744 ± 0.297	2.489 ± 0.241	2.318 ± 0.161	2.216 ± 0.197	2.211 ± 0.263	0.745 ± 0.056	0.574 ± 0.136	0.472 ± 0.1	0.467 ± 0.034

Values are mean  ±  SD; * *p*  <  0.05 at the Student’s *t*-test. * *p*  <  0.05, significantly different from control, *n*  =  3.

## Data Availability

Data is contained within the article and Supplementary Materials.

## Supplementary Materials

The following are available online at https://www.mdpi.com/article/10.3390/ph15020114/s1, Figure S1: HPLC-DAD chromatograms of reference, Figure S2: Mass spectra of the formula F, Figure S3: HPLC-DAD chromatogram of reference compounds.
