# Supplementary material for: Antioxidant–Anti-Inflammatory Evaluation of a Polyherbal Formula
Source: Pharmaceuticals (Basel). 2022 Jan 18;15(2):114. doi: 10.3390/ph15020114 (PMC8874475; doi:10.3390/ph15020114)
Supplement: Supplementary file 1 [file pharmaceuticals-15-00114-s001.zip › pharmaceuticals-1475152-supplementary.pdf]

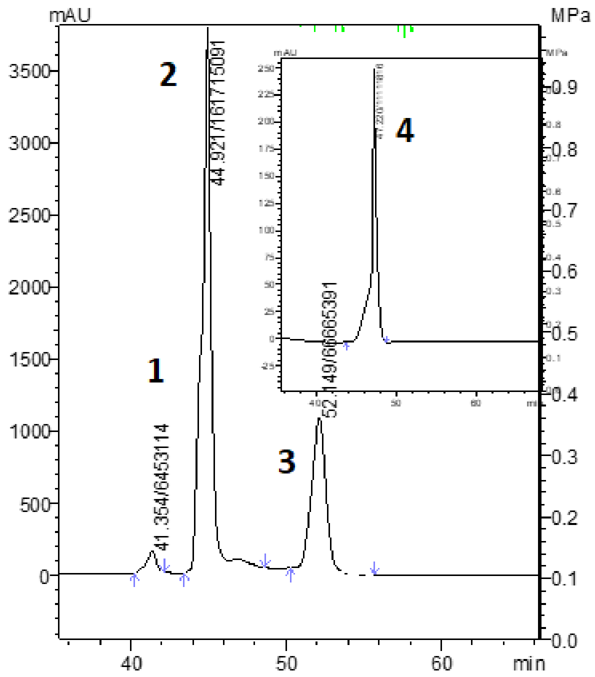

Figure S1. HPLC-DAD chromatograms of reference compounds – 1. Salicin; 2. Acteoside; 3. Unknown; 4. Oleanolic acid (inset)

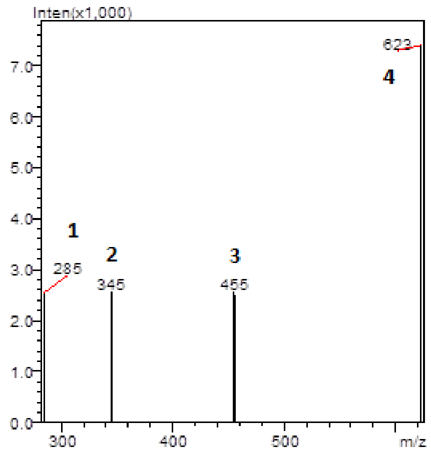

Figure S2. Mass spectra of the formula F – 1. Salicin; 2. Aucubin; 3. Oleanolic acid; 4. Acteoside

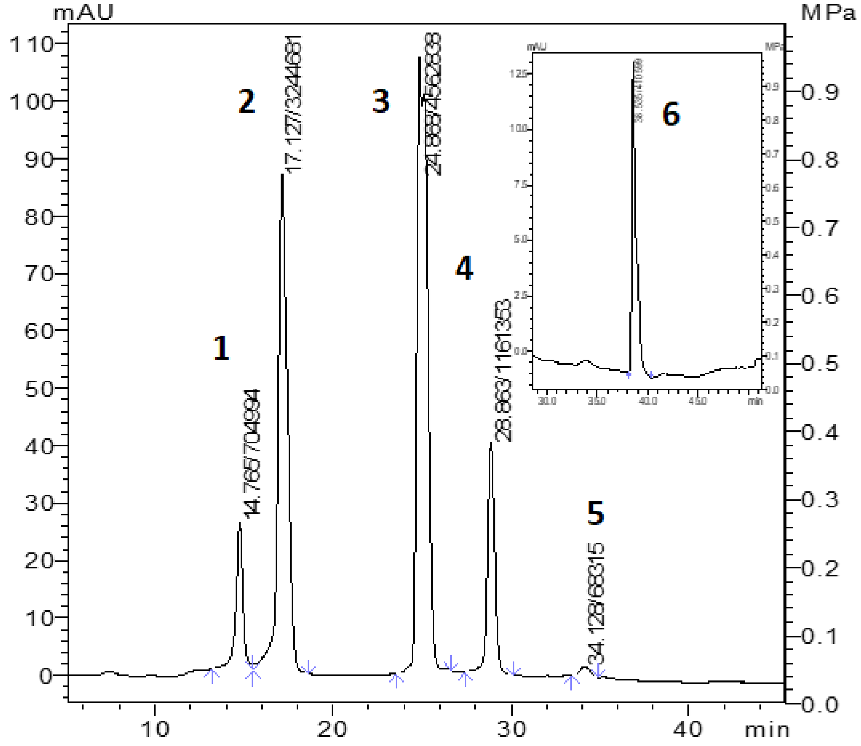

Figure S3. HPLC-DAD chromatogram of reference compounds – 1. Chlorogenic acid; 2. Caffeic acid; 3. Ferulic acid; 4. Rosmarinic acid; 5. Quercetin; 6. Apigenin (inset)
